# Facing the challenges to shorten the diagnostic odyssey: first Whole Genome Sequencing experience of a Colombian cohort with suspected rare diseases

**DOI:** 10.1038/s41431-024-01609-8

**Published:** 2024-06-22

**Authors:** Harvy Mauricio Velasco, Aida Bertoli-Avella, Carolina Jaramillo Jaramillo, Danny Styvens Cardona, Leonel Andrés González, Melisa Naranjo Vanegas, Juan Pablo Valencia Arango, Cesar Augusto Buitrago, Jorge Alberto Gutiérrez González, Jonas Marcello, Peter Bauer, Juliana Espinosa Moncada

**Affiliations:** 1 Personalized Medicine Group, Unidad de Bioentendimiento, Bioscience Center, Ayudas Diagnósticas SURA, Medellín, Colombia; 2 CENTOGENE GmbH, Rostock, Germany; 3 Sura Omics Science Center, Unidad de Bioentendimiento, Bioscience Center, Ayudas Diagnósticas SURA, Medellín, Colombia; 4 Data Science Department, Bioscience Center, Ayudas Diagnósticas SURA, Medellín, Colombia; 5 Medical Imaging & AI in Health SURA, Bioscience Center, Ayudas Diagnósticas SURA, Medellín, Colombia; 6 University Hospital of Rostock, Hematology, Oncology, and Palliative Medicine, Rostock, Germany

**Keywords:** Genetics research, Translational research

## Abstract

Exome and genome sequencing (ES/GS) are routinely used for the diagnosis of genetic diseases in developed countries. However, their implementation is limited in countries from Latin America. We aimed to describe the results of GS in patients with suspected rare genetic diseases in Colombia. We studied 501 patients from 22 healthcare sites from January to December 2022. GS was performed in the index cases using dried blood spots on filtercards. Ancestry analysis was performed under iAdmix. Multiomic testing was performed when needed (biomarker, enzymatic activity, RNA-seq). All tests were performed at an accredited genetic laboratory. Ethnicity prediction data confirmed that 401 patients (80%) were mainly of Amerindian origin. A genetic diagnosis was established for 142 patients with a 28.3% diagnostic yield. The highest diagnostic yield was achieved for pathologies with a metabolic component and syndromic disorders (*p* < 0.001). Young children had a median of 1 year of diagnostic odyssey, while the median time for adults was significantly longer (15 years). Patients with genetic syndromes have spent more than 75% of their life without a diagnosis, while for patients with neurologic and neuromuscular diseases, the time of the diagnostic odyssey tended to decrease with age. Previous testing, specifically karyotyping or chromosomal microarray were significantly associated with a longer time to reach a definitive diagnosis (*p* < 0.01). Furthermore, one out of five patients that had an ES before could be diagnosed by GS. The Colombian genome project is the first Latin American study reporting the experience of systematic use of diagnostic GS in rare diseases.

## Introduction

Rare diseases (RD) comprise about 5000 to 8000 medical disorders, each of which affects a small number of patients, but collectively afflict millions [1]. Most of them have a pediatric onset (69.9%) and are responsible for 25% of all pediatric hospitalizations [2]. In Latin America (LATAM) about 7% of the population (~40–50 million people), are living with a rare disease, posing a major public health challenge for these countries [3]. Regardless of the geography, RD share elements such as chronicity, severity, unknown natural history, onerous management, compromised quality of life and difficulties in establishing the diagnosis. Usually, the rarity of the disease is associated with a delay or even lack of diagnosis [4].

The “diagnostic odyssey” describes the journey of the patient and family members toward finding a diagnosis, which may begin prenatally, during the neonatal period, in infancy, but also in adulthood. Shortening or ending the odyssey has important clinical, psychosocial, and economic benefits [5, 6].

Colombia is the third most populous country in LATAM with 52 million inhabitants in an area of 2,070,408 km^2^ [7], divided into five regions (Andean, Pacific, Caribbean, Amazonía and Orinoquía). Colombia has a RD incidence of less than 1:5000 [8]. The census of RD in Colombia was initiated in 2016, with about 12,000 new patients registered every year. It is thought that 10 to 30% of them have a genetic component [9, 10]. Since the last decade, changes in the Colombian health coverage services have allowed a wider utilization of Sanger sequencing, MLPA (multiplex ligation-dependent probe amplification), chromosomal microarrays (CMA) and Next-generation sequencing (NGS) methodologies. Furthermore, from 2015 exome sequencing (ES) was implemented as a useful tool to achieve genetic diagnoses, establish therapeutic management, and provide genetic counseling to patients and families with RD [10]. It is recognized that the diagnostic yield of ES is between 20 to 30%, being higher in critically ill patients or with multiple malformations [11, 12]. The American College of Medical Genetics and Genomics guidelines (ACMG) recommend ES as a first tier test in pediatric patients with congenital anomalies or intellectual disability [13].

Genome sequencing (GS) could offer a higher diagnostic yield [14], given its technical advantages, with even coverage which includes the non-coding regions, better detection of copy number variants (CNV), structural variants (SV) and even the detection of repeat expansions [14]. GS has been particularly useful in patients with intellectual disability and malformations (40–55%) [15]. However, there is limited data on the use of ES for the diagnosis of RD in LATAM [16–19]. Furthermore, only a few initiatives are known that applied GS such as the Rare Genomes Project [20], the Odyssey Project, the Brazilian Genomes Project (https://www.genomasraros.com/projeto/), and the 80 Plus Project. Apart from the information provided by the Rare Genomes Project, with a preliminary diagnostic yield of 35.7%, we have no further evidence of the diagnostic or clinical utility of GS in LATAM [21]. In this paper, we describe these characteristics, and, in addition, we analyzed the diagnostic performance of the test and the diagnostic odyssey in the largest Colombian cohort of patients with suspected rare diseases reported until now.

## Materials and methods

### Study design and settings

We evaluated 501 participants that were referred for genetic testing by specialized physicians and clinical geneticists at a healthcare provider in Colombia, from January to December of 2022. The study included 22 healthcare centers in Andina, Pacific and Caribbean regions. A retrospective analysis was made to describe GS results for the 501 patients (Supplementary Appendix 1).

### Participants

All eligible patients were asked to participate in the study and informed consent for GS testing and genetic testing was given by adult patients, parents of minors, or caregivers, respectively. Inclusion criteria included all consecutive patients with a suspected RD. Exclusion criteria are defined in Supplementary Appendix 2. No follow-up of patients was done. Data regarding family history, consanguinity, disease onset, motive of referral and previous genetic testing were extracted from our database and curated individually by medical geneticists and molecular biologists.

### Variables

All variables were collected from primary sources within the 22 participating healthcare centers. Four main categories were considered: demographic variables, clinical variables, genetic variables, and related to patient evolution and outcomes (treatment, genetic counseling).

### Clinical data

The clinical information provided by referring clinicians was converted into Human phenotype ontology (HPO) terms by a dedicated team of scientists at the Bioscience Center, Sura Omics Science Center in SURA Colombia and CENTOGENE in Germany. During this process, relevant information was curated using HPO terms and registered in the laboratory management system (LIMS) and CENTOGENE Biodatabank. This clinical information was used to assess the relevance of the identified variants.

### Genome sequencing (GS) and bioinformatic pipeline

DNA was extracted from dried blood spots on filters (CentoCard ®) using standard column-based methods. GS was performed as described before [22]. In brief, genomic DNA was cleaved enzymatically, and libraries were generated by PCR with Illumina compatible adapters. The libraries were paired end sequenced on an Illumina platform to yield an average coverage depth of ~30x. An in-house bioinformatics pipeline, including read alignment to GRCh37/hg19 genome assembly and revised Cambridge Reference Sequence (rCRS) of the Human Mitochondrial DNA (NC_012920), variant calling, annotation, and comprehensive variant filtering is applied. Copy number variation (CNV) calling is based on the DRAGEN pipeline from Illumina. All variants with minor allele frequency (MAF) of less than 1% in gnomAD database, and disease-causing variants reported in HGMD®, in ClinVar or in CentoMD® were evaluated.

Although the evaluation was focused on coding exons and flanking intronic regions, the complete gene region was interrogated for candidate variants with plausible association to the phenotype. All potential mode of inheritance were considered (autosomal recessive, autosomal dominant, X-linked dominant and recessive, mitochondrial) along with variant features such as observed zygosity, frequency in external and internal databases, phenotype of individuals with the same variant/zygosity, in silico predictions, gene-disease validity according to ClinGen guidelines, known disease mechanism, and variant classification in CENTOGENE and external databases. Mitochondrial variants are reported for heteroplasmy levels of 15% or higher. Filtering and variant evaluation is performed with a tool developed at CENTOGENE. Variants with a minimum read depth of 10 reads, and variant allele fraction (>25%) were considered, along with a general quality score >100. Details of the quality score calculation were published before [23]. Variants with allele fraction <25% might indicate mosaicism or sequencing artifacts. Visual inspection of each variant considered for reporting was done (BAM files visualized using the IGV browser) [24]. Consequently, a technical specificity, i.e., accuracy of the observed genotypes of >99.9% for all reported variants is achieved.

### Biochemical testing

Biochemical testing assays from dried blood spots (DBS) were performed to clarify the effect of genetic variants with determination of the corresponding enzymatic activity and/or biomarker concentration. The enzymatic activities were determined either by fluorimetry or liquid chromatography coupled with mass spectrometry in DBS. The quantification of the biomarkers was performed in DBS using mass spectrometry (a full list of the tests can be found in [25]).

### RNA-seq

A protocol was developed to perform RNA-seq using RNA extracted from filter card DBS, library preparation and bioinformatic pipeline analysis. Splicing patterns in cases were inspected using IGV interactive program and adding three independent controls. Relative gene expression was calculated using housekeeping genes and compared against controls.

### Variants classification and reporting

Variants are classified into five classes (pathogenic, likely pathogenic, variants of uncertain significance (VUS), likely benign, and benign) along ACMG/AMP SVI guidelines for classification of genetic variants. All relevant variants related to the phenotype of the patient were reported [26]. In addition, upon signed consent, pathogenic and likely pathogenic variants not associated with the patient’s disease or symptoms but medically actionable (Secondary Findings) are reported in a dedicated section (73 selected genes according to ACMG guidelines) [27]. Furthermore, in the carriership section, we report pathogenic and likely pathogenic sequence variants in known genes associated with severe and early-onset autosomal recessive and X-linked disorders regardless of the current phenotype. The selection of over 2000 genes was carefully curated to reflect up-to-date scientific and medical knowledge (e.g., includes genes from the ACMG carrier screening guidelines [28].

### Analytic strategy

A descriptive analysis was performed, which includes summary measures such as medians, percentiles, and proportions. Associations were statistically qualified using Fisher´s exact test or Chi-square, as appropriate. *p* values less than 0.05 were considered significant. The Mann–Whitney and Kruskal–Wallis *U* test were also used to determine the difference between groups; the Shapiro–Wilk test was used to assess the normality of the variables.

Ethnicity admixtures were predicted using the iAdmix software (*iAdmix is released under the MIT license) [29]. A model with 25 distinct ethnicities was trained using a research dataset from previous whole genome analysis at CENTOGENE. During the ethnicity prediction, we obtain a maximum likelihood estimate of the global admixture proportions using the Broyden-Fletcher-Goldfarb-Shanno (BFGS) optimization algorithm for each of the 25 ethnicities. The Top 3 ethnicities for every patient are used for subsequent analysis.

All the statistical analyzes were developed in the statistical software R, version 4.2.0.

## Results

We included 501 index cases with suspected genetic diseases, without a conclusive clinical diagnosis. Many patients had had previous genetic testing such as karyotype (24%), ES (24%) and CMA (22%). Over half of the patients were children or preadolescents (52%), 16% were adolescents and 32% were adults. The median age at onset of symptoms was 1 year (0–12). In a small number of patients (8.9%) parental consanguinity was reported, while many of them mentioned positive family history (44%) (Table 1).

**Table 1:** Demographic and clinical characteristics of the 501 patients from the Colombian rare genomes project.

Cohort characteristics	Total *N* = 501^a^
Sex	
Male	252 (50%)
Female	249 (50%)
Age	12 (6, 29)
Age of onset symptoms	1 (0, 12)
Age at diagnosis	12 (6, 29)
Age groups	
Group 1 (0–6 years)	147 (29%)
Group 2 (6–12 years)	112 (22%)
Group 3 (12–19 years)	81 (16%)
Group 4 (19–45 years)	90 (18%)
Group 5 (45–86 years)	71 (14%)
Parental consanguinity	39 (8.9%)
Family positive history	219 (44%)
HPO terms	5 (3, 6)
Number of taxonomies	2 (2, 3)
Number of genetic studies	1 (0, 2)
Birth area	
Andean region	457 (91.2%)
Pacific region	27 (5.4%)
Caribbean region	15 (3%)
Orinoquia region	2 (0.4%)

^a^*n* (%), median (Q1, Q3).

Pearson’s Chi-squared tests; Fisher’s exact test.

**Fig. 1:**
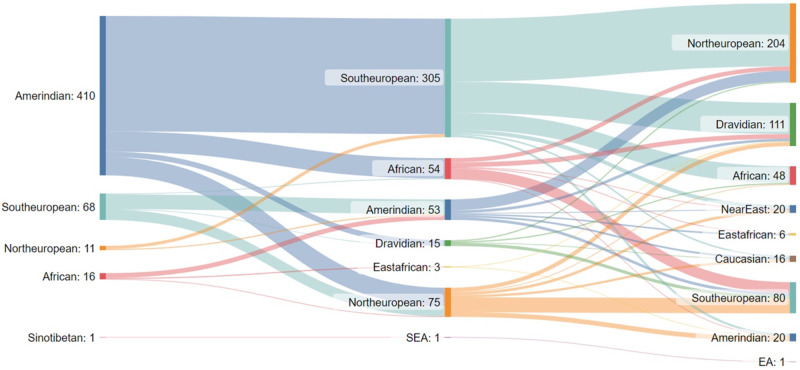
Dendogram of cohort ancestry. Dendrogram representing the ancestry analysis results from the 501 patients included in this study. The results illustrate the preponderance of an Amerindian component close to 81% (410 patients), followed by South European with 16% (305 patients). The ancestry predictions are shown as number of patients, with TOP 3 predictions shown. First prediction: left side, second prediction at the middle and third prediction at the right side of the diagram.

Most of the patients originated from the province of Antioquia (Andean/Northwestern Region) due to the health insurance coverage in this region (SURA health insurance company). The analysis highlighted a preponderance of Amerindian/American component close to 81% (*n* = 410 patients), followed by European with 16% (mainly South European, *n* = 305). Only 79 patients had European ethnicity as first prediction, and 16 patients African (Fig. 1).

**Fig. 2:**
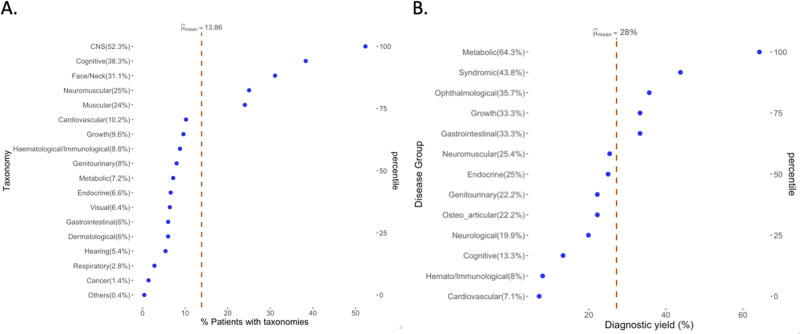
Taxonomy and Diagnosis of the Colombian Rare Project. **A** Diverse taxonomy groups (in percentages) among 501 patients in the Colombian rare genomes project. Taxonomy groups were formed according to the main signs and symptoms of the patients. The dotted orange line reflects the mean of the percentages. The distribution of each taxonomy was analyzed independently. Disorders of the CNS (52.3%) and of the cognitive functions (38.3%) were the most frequently detected. **B** Percentage of positivity by disease group. The *x*-axis describes the percentage of the overall diagnostic yield (dashed orange line) and of each disease group independently. The *y*-axis describes the disease groups. The highest diagnostic yield was achieved for metabolic disorders (64.3%), followed by syndromic disorders (43.8%).

Phenotypic analysis was conducted using HPO (Human Phenotype Ontology) terms, with an average of 5 terms per patient. To determine the most affected taxonomic group, signs and symptoms with the highest clinical impact were considered. The current cohort of patients exhibited an average of 2 affected taxonomies. CNS structural abnormalities were observed in 262 (52%) patients, cognitive involvement was detected in 192 patients (38%), facial dysmorphisms were identified in 156 patients (31%), and neuromuscular disorders were present in 125 patients (25%) (Fig. 2A).

### Molecular findings

**Fig. 3:**
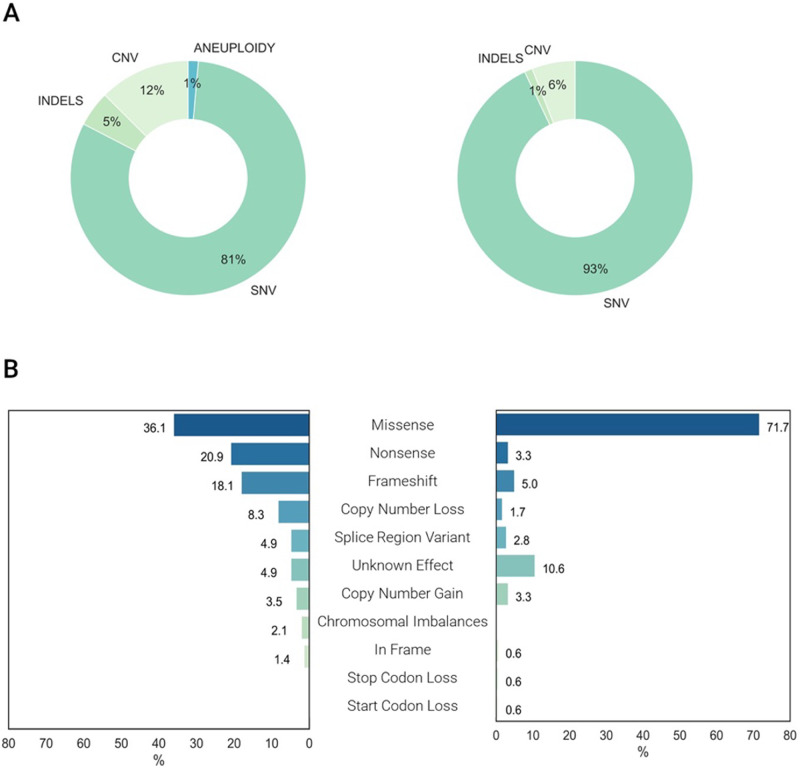
Characteristics of variant found in the Colombian Cohort with RD. **A** This figure shows the type of variants classified as P/PL (left) and VUS (right) in percentages. **B** The bar graph shows the type of variants according to their molecular impact among P/LP variants (left) and VUS (right), in percentages. As expected, there were more CNVs and indels among P/LP variants than among VUS (12% and 5% vs. 6% and 1%, respectively), with a predominance of nonsense and frameshift variants in the P/LP group compared to the VUS group (20.9% and 18.1% vs. 3.3% and 5%, respectively). The VUS group was composed mainly by missense and variants with unknown effect (e.g. noncoding variants). SNV single nucleotide variant, INDEL insertion/deletion variant, CNV copy number variant.

Clinically relevant variants were reported in 237 genes with 144 pathogenic/likely (P/LP) pathogenic variants. Several of these P/LP variants were repeatedly detected in genes such as *CYP21A2* (5 patients), *SCN1A*, *GBA* and *FKBP14* (4 patients each) (Supplementary Appendix 3). Of the variants classified as P/LP, 96% were in coding regions, whereas 4% were noncoding variants in *RMRP*, *POLR3A*, *NPC1, UGT1A1*, and *CYP21A2*. Most of the variants corresponded to SNVs (81%), followed by CNVs (12%), small indels (5%) and aneuploidies (1%) (Fig. 3A). Missense variants were the most reported type in this dataset (Fig. 3B). When comparing the molecular effect between the P/LP and VUS group, there were significant differences (*p* < 0.01), with more missense variants reported in the VUS group (71% vs. 36.1%) and more loss of function (LoF) variants in the P/LP group (Fig. 3B), as expected.

**Fig. 4:**
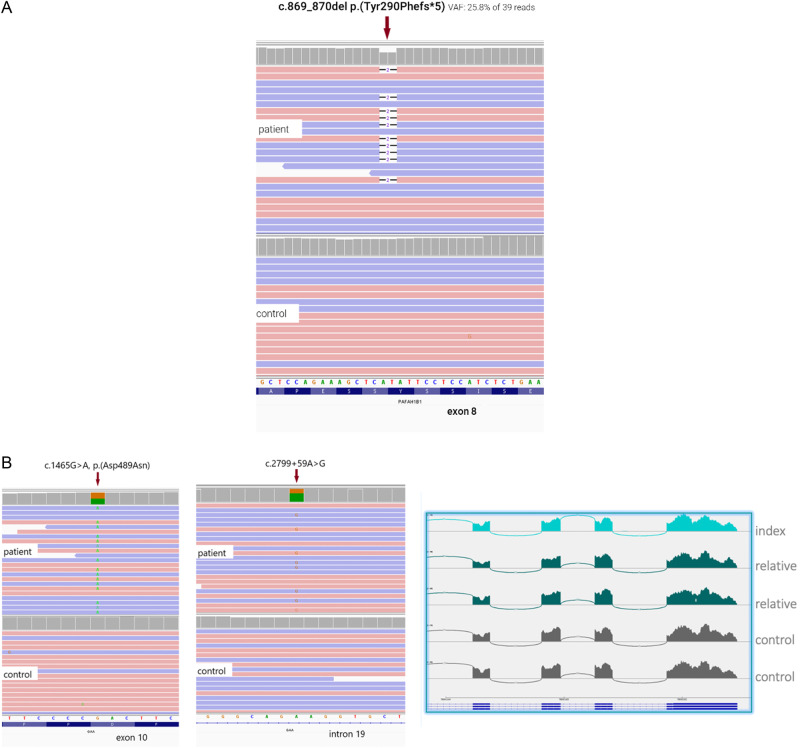
Molecular description of interest cases patients with RD in the cohort. **A** Detection of mosaic variant in a 11-year-old patient in the *PAFAH1B1* gene. A pathogenic variant was detected in 25.8% of the reads (IGV browser). This result confirmed the diagnosis of lissencephaly type 1, autosomal dominant. **B** IGV image showing two variants in the *GAA* gene (in compound heterozygosity in a 14-year-old patient) The first variant is pathogenic c.1465G > A p. Asp489Asn and, the second is a variant of uncertain significance c.2799+59A > G. The transcriptomic study showed no splicing alterations between exons 18 and 19, thus, the second variant remains of uncertain significance.

Most of the diagnosed diseases had a mode of inheritance compatible with autosomal dominant inheritance (71, 50.4%), followed by autosomal recessive (51, 36.2%), X-linked (14, 9.9%), aneuploidies (4, 2.8%) and, with mitochondrial inheritance (1, 0.7%). A mosaic case was also detected corresponding to an 11-year-old patient with a molecular diagnosis of lissencephaly and subcortical band heterotopia (OMIM: *601545, patient GM86) with a mosaic *PAFAH1B1* variant NM_000430.3:c.869_870del p. (Tyr290Phefs*5) detected in 25.8% of the reads (Fig. 4A).

In selected cases, the clinical significance of the detected variants was further investigated by additional testing using the same sample provided. In a male patient (GM214) with neurodevelopmental delay, seizures, hepatosplenomegaly and tetraparesis, we detected two heterozygous P/LP variants in *NPC1*. The biomarker PPCS (C24H50O7N2P—legacy name lyso SM-509) was pathologically increased, which supported the diagnosis of Nieman-Pick disease type C1 in the patient. The second patient (GM378) was a 4-year-old male, with bilateral coxa valga, encephalopathy, dysphagia, periventricular leukomalacia, dental malocclusion, developmental regression, and growth delay. We detected a hemizygous VUS in the gene *IDS* NM_000202.5:c.1477C > T p. (Arg493Cys). Consecutively, the enzymatic activity of iduronate sulfatase was found pathologically decreased, supporting the diagnosis of Mucopolysaccharidosis II in the patient. In a third patient (GM176), a 14-year-old male presented with weakness in lower extremities since the age of 12 years. We detected two variants in *GAA*, a pathogenic (NM_000152.3:c.1465G > A p. (Asp489Asn)) and VUS (NM_000152.3:c.2799+59A > G) in compound heterozygous state. Further testing detected a mildly reduced acid alpha-glucosidase activity. Screening for additional heterozygote individuals for any of the detected variants revealed that the pathogenic variant was leading to approximately a 60% reduction of the enzymatic activity, while individuals with the heterozygous intronic VUS presented normal enzyme activity. Furthermore, RNA-seq analysis excluded an abnormal splicing of *GAA*. Taken together, the use of multiomic analysis supported a non-causal role for the c.2799+59A > G variant, making the diagnosis of Pompe disease unlikely (Fig. 4B).

### Diagnostic yield

A molecular diagnosis could be defined for 142 (28.3%) index cases. The highest diagnostic yield was achieved for pathologies with a metabolic component (*n* = 18, 64.3%, *p* < 0.001), followed by syndromic disorders (*n* = 56, 43.8%, *p* < 0.001), neurological abnormalities such as neuromuscular (*n* = 17, 25.4%), neurological (*n* = 28, 19.9%) and intellectual disability (*n* = 6, 13.3%). Diseases with a major cardiovascular or hemato-immunological component had lower diagnostic yields in our cohort (*p* < 0.05) (Fig. 2B).

A total of 119 patients (24%) from this cohort had previous negative or inconclusive ES studies and of these, 23 patients (19.3%) received a positive diagnosis with GS. Among these there were two SNVs located in noncoding regions (GM79, *RMRP*, associated with cartilage-hair hypoplasia and GM275, *HSD17B4*, associated with Perrault syndrome). There were also four large CNVs: three of them were copy-number losses (GM43, *CLCNKB* gene; GM146, *APTX* gene in homozygosis and GM143, *BPTF* in heterozygosis) and one case of copy-number gain affecting the *DMD* gene in patient GM394 (Table 2).

**Table 2:** Description of relevant diagnoses with their respective pathogenic and probably pathogenic variants.

Noncoding findings (patients with inconclusive/negative ES)							
ID	Age	Sex	Variant	Zygosity		Classification	Associated phenotype
GM79^b^	51	Female	*RMRP* NR_003051.3:n.196C > T		Het	LP	Cartilage-hair hypoplasia, AR (OMIM: 250250)
			*RMRP* NR_003051.3: c.-9_10insTCGAAGTGTCTCATCA		Het	VUS	
GM116	49	Female	*POLR3A* NM_007055.3:c.3781G > T p.(Glu1261*)		Het	LP	Leukodystrophy, hypomyelinating, 7, with or without oligodontia and/or hypogonadotropic hypogonadism, AR (OMIM: 607694)
			*POLR3A* NM_007055.3:c.1909+22G > A		Het	P	
GM214	18	Male	*NPC1* NM_000271.4:c.1554-1009G > A		Het	P	Niemann-Pick disease, type C1, AR (OMIM: 257220)
			*NPC1*: NM_000271.4:c.1780_1782del p.(Tyr594del)		Het	LP	
GM275	31	Female	*HSD17B4* NM_001199291.1: c.1512+1866C > T		Het	LP	Perrault syndrome, AR (OMIM: 233400)
			*HSD17B4* NM_001199291.1:c.662C > T p.(Ala221Val)		Het	LP	
GM418	17	Female	*UGT1A1* NM_000463.2:c.-56_-55insAT		Hom	LP	Gilbert syndrome, AR (OMIM: 143500)
			*UGT1A1* NM_000463.2:c.-3275T > G		Hom	LP	
GM470	24	Female	*CYP21A2* NM_000500.5:c.293-13C > G		Hom	P	Adrenal hyperplasia, congenital, due to 21-hydroxylase deficiency, AR (OMIM: 201910)
CNVs findings							
GM43	3	Male	NC_000001.10:g.(16360482_16383317)del *CLCNKB* NM_000085.4 exons 1-18 *CLCNKA* NM_004070.3 downstream		Hom	P	Bartter syndrome, type 3, AR (OMIM: 607364)
GM143	3	Male	NC_000017.10:g.(65955641-65983882)del *BPTF* NM_004459.7 exons 26-30		Het	LP	Neurodevelopmental disorder with dysmorphic facies and distal limb anomalies, AD (OMIM: 617755)
GM146	35	Male	NC_000009.11:g.(32985581_32986746)del *APTX* NM_175073.3 exon 6		Hom	LP	Ataxia, early-onset, with oculomotor apraxia and hypoalbuminemia; AR (OMIM: 208920)
GM394	6	Male	NC_000023.10:g.(33150119_33310797)dup *DMD* NM_004006.3 exon 1		Het	LP	Becker muscular dystrophy; LX (OMIM:300376), Duchenne muscular dystrophy; LX (OMIM: 310200)
Secondary findings in ACMG genes^a^							
GM255	4	Male	*TTR* NM_000371.3:c.424G > A p.(Val142Ile)		Het	P	Amyloidosis, hereditary, transthyretin-related; AD (OMIM:105210)
GM294	86	Female	*RYR1* NM_000540.2:c.848A > G p.(His283Arg)		Het	LP	Malignant hyperthermia susceptibility 1; AD (OMIM: 145600)
GM299	15	Female	*HFE* NM_000410.3:c.845G > A p.(Cys282Tyr)		Hom	P	Hemochromatosis; AR (OMIM: 235200)
GM305	9	Female	*BTD* NM_001281723.2:c.1336G > C p.(Asp446His)		Hom	P	Biotinidase deficiency; AR (OMIM: 253260)
GM413	6	Male	*TTR* NM_000371.3:c.424G > A p.(Val142Ile)		Het	P	Amyloidosis, hereditary, transthyretin-related; AD (OMIM:105210)
GM424	2	Male	*LDLR* NM_000527.2:c.502G > A p.(Asp168Asn)		Het	P	Hypercholesterolemia, familial, 1; AD, AR (OMIM: 143892)
			*PMS2* NM_001322014.1:c.989-2A > G		Het	LP	Lynch syndrome 4; AD (OMIM: 614337)
GM441	24	Male	*KCNH2* NM_000238.3:c.1262C > T p.(Thr421Met)		Het	LP	Long QT syndrome 2; AD (OMIM: 613688)
GM476	6	Female	*TTN* NM_001267550.1:c.49970G > A p.(Trp16657*)		Het	LP	Cardiomyopathy, dilated, 1G; AD (OMIM:604145)
GM482	6	Male	*PMS2* NM_001322014.1:c.400C > T p.(Arg134*)		Het	P	Lynch syndrome 4; AD (OMIM: 614337)
GM487	39	Female	*APOB* NM_000384.2:c.6557_6560del p.(Lys2186Ilefs*8)		Het	LP	Hypercholesterolemia, familial, 2; AD (OMIM: 144010)
		Patients with double diagnosis					
GM81	1	Female	*SCN1A* AB093548.1:c.4126_4139del p.(Cys1376Asnfs*3)		Het	LP	Generalized epilepsy with febrile seizures plus, type 2; AD, Febrile seizures, familial, 3A; AD (OMIM: 604403). Dravet syndrome; AD (OMIM: 607208)
			*HBB* NM_000518.4:c.20A > T p.(Glu7Val)		Het	P	Sickle cell anemia; AR (OMIM:603903) Patient presented chronic anemia
GM319	20	Female	*VWF* NM_000552.4:c.5455+1G > A		Het	LP	Von Willebrand disease; AD, AR (OMIM: 193400, 277480, 613554) Patient with symptomatic bleeding.
			seq[GRCh37] 4p16.3p16.1(68726_8873264)x3		Het	LP	4p16.3 microduplication syndrome
			seq[GRCh37] 8p23.3p23.1(155255_7037109)x1		Het	P	
GM347	9	Female	*SLC2A1* NM_006516.2: c.850C > G p.(Leu284Val)		Het	LP	GLUT1 deficiency syndrome 1, infantile onset, severe; AD, AR (OMIM: 606777)
			arr[GRCh37] Xq22.3q28 (106897552_155236204) x1		Het	P	Partial monosomy of the Xq chromosome
GM467^c^	32	Female	*PPP1R21* NM_001135629.2: c.-19_7del		Hom	VUS	Neurodevelopmental disorder with hypotonia, facial dysmorphism, and brain abnormalities; AR (OMIM: 619383)
			*HBB* NM_000518.4: c.20A > T p. (Glu7Val)		Het	P	Sickle cell anemia; AR (OMIM:603903). Patient with symptomatic hemolytic anemia

*Het* heterozygous, *Hom* homozygous, *LP* likely pathogenic, *P* pathogenic, *AD* autosomal dominant, *AR* autosomal recessive, *CNVs* copy number variants.

^a^Findings based on ACMG recommendations (ACMG SF v3.1 list for reporting of secondary findings in clinical exome and genome sequencing [27].

^b^The variant is found in the promoter region between the TATA box and the transcriptional start site, where similar insertions and duplications have been reported in patients with cartilage-hair hypoplasia spectrum disorders—anauxetic dysplasia. Experimental evidence indicates that these insertions would silence *RMRP* transcription; together with the retrospective clinical correlation, this variant is considered as likely causative.

^c^Retrospective clinical evaluation indicated that this variant is likely explaining the phenotype of the patient.

We analyzed features such as parental consanguinity, positive family history, age at onset of symptoms, number of HPO terms, among other features. Only the total number of taxonomies suggested a statistical relationship to the final diagnostic result, with patients with more taxonomies (3 or more) having a positive GS result (Supplementary Table 1). A multiorgan involvement, as reflected in a higher number of affected taxonomic groups per patient, might be indicative of an underlining genetic disorder. This can explain the higher diagnostic yield among patients with higher number of affected taxonomies (Supplementary Table 1).

In addition, the carriership status for P/LP variants with relevance for family planning were analyzed for all patients/families (upon consent, see Methods section). Sixty-one percent of the patients analyzed (308/501) had at least one P/LP variant in any of 162 reported genes (heterozygous carrier status). The most frequently reported variants were in the following genes: *BTD* (*n* = 33, 7%), *GJB2* (*n* = 17, 4%), *CFTR* (*n* = 13, 3%), *CBS* (*n* = 11, 2%), *BCHE* (*n* = 10, 2%), *ALG12* (*n* = 9, 2%, *HBB* (*n* = 8, 2%), *DPYD* (*n* = 8, 2%), *POLR3A* (*n* = 8, 2%). As for secondary findings, according to the recommended list of genes of the ACMG guidelines [30], we found that 11 patients (2%) presented relevant variants (Table 2).

### Diagnostic odyssey

In 142 positive cases, we examined the time elapsed between symptom onset and genetic diagnosis. As expected, the diagnostic odyssey was longer in older patients, with a median of 1 year for young children (0–5 years) versus a median of 15 years in adolescents and adult patients (13–35- and 36–86-year groups). The most significant differences were found between extreme age groups (0–5 years and 36–86 years, *p* value < 0.001, Supplementary Appendix 4). Thus, to facilitate and allow further comparations among patients, we corrected for the patients’ age and normalized the odyssey time by age. Note that this is a relative measure of the interval of time that a person lived without a diagnosis (diagnostic odyssey).

**Fig. 5:**
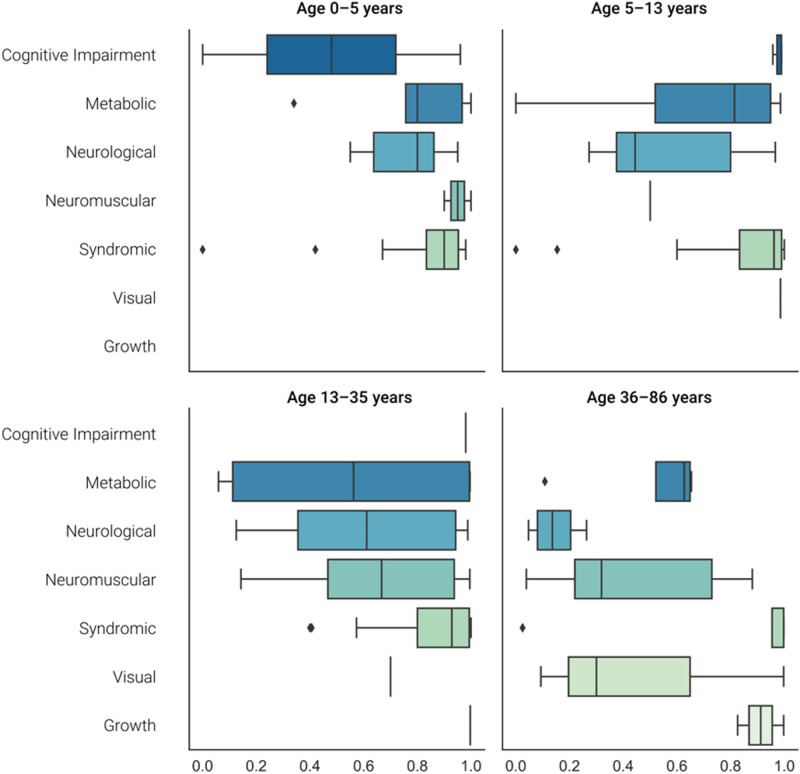
Standardized diagnostic odyssey (*X*-axis) according to age ranges and groups of pathologies (*Y*-axis). Each quadrant of the figure shows a distribution for each age group. Children between 0–5 years of age with neurological diseases spent ~80% of their life in the odyssey, while children between 5–13 years spent 40%.

Then, we observed interesting differences in the diagnostic odyssey according to the pathology and age ranges. Patients with syndromic disorders have spent more than 75% of their life without a diagnosis, while for patients with neurologic and neuromuscular diseases, the time of the odyssey tended to decrease with age (inversely related, Fig. 5).

Odyssey time intervals were also analyzed according to taxonomy groups. The diagnostic odyssey was longer for patients with taxonomy associated with face and neck abnormalities (*p* = 0.003) and with growth disorders (*p* = 0.002), while shorter time intervals were observed for patients presenting neuromuscular abnormalities (*p* = 0.045) (Supplementary Appendix 5).

We then analyzed the possible relationship between previous genetic testing and the odyssey time. Having karyotype or CMA testing before GS was associated with a longer time to reach a definitive diagnosis (*p* < 0.01). Having a previous ES did not show any significant relationship. No statistical differences in odyssey times were observed when analyzing other variables such as parental consanguinity and positive family history (*p* > 0.05).

## Discussion

In this work we describe the results of GS applied to a clinically heterogeneous cohort of 501 patients with suspected RD from Colombia. Of note, patients with clinical entities that could be easily diagnosed by ES or gene panels were not included, given the well-known diagnostic performance of these tests in patients with hereditary cancer, easily characterized dysmorphic syndromes, among others.

The patients from this cohort were presented with 970 HPO terms, 18 taxonomies, and 196 clinically suspected diseases (ICD10) [4, 9]. A recent Latin American study of 103 Chilean patients with heterogeneous genetic phenotypes analyzed with ES showed an average of 6 compromised systems [31].

By using a dedicated ethnicity prediction tool, we show that most of the patients included in the study have an Amerindian (native American) origin which has been underrepresented in public databases and other NGS studies [14, 21]. Importantly, this is the first LATAM study reporting the experience of systematic use of diagnostic GS. Efforts to include diverse populations in genomic studies are essential for advancing precision medicine and developing interventions across different ethnic backgrounds.

In this cohort, the diagnostic yield was 28.3%, which is within the known range of 22–50% reported in other studies [13]. Importantly, a higher diagnostic yield of 64.3% was observed among patients with suspected metabolic disorders and a yield of 43.8% was observed among patients with syndromic involvement, supporting the indication of GS as a preferred testing method or first tier for these patient groups. The high diagnostic yield obtained for metabolic disorders might have two main reasons. Firstly, the lack of early diagnosis (e.g., by newborn screening) in Colombia. Currently, newborn screening in Colombia only includes congenital hypothyroidism. Further efforts are required to reduce the gap in the diagnosis of metabolic pathologies. Secondly, the application of multi-omics (e.g., biomarkers, enzymatic activity measurements), and/or RNA-seq allowed us to further understand the relevance of the detected variants, which is especially useful in metabolic disorders.

There are few GS/ES studies reported in LATAM. In 2019, a study based on 60 Mexican patients with suspected genetic diseases explored the use of GS as first-tier genetic test [32]. Another study referred the experience with ES in Brazil, which describes a diagnostic yield of 31.6% which was driven by the prenatal samples included (67% diagnostic rate), and children younger than 1 year (44% diagnostic rate) [19]. The difference in diagnostic yield with the current study could be due to the age of the patients included. Prenatal samples were not included in our study, with a median patient age of 12 years [4, 9].

Although in most cases, the search for the genetic cause of diseases is focused on the coding regions, the study of non-coding regions is becoming increasingly relevant, variants located on introns and regulatory regions could explain an important percentage of diseases [33]. For example, a recent RNA-seq study of 15 patients clinically diagnosed with Neurofibromatosis type 1 but negative NF1 testing, identified that 10 out of the 15 patients presented noncoding changes including deep intronic variants, transposon insertions causing noncanonical splicing, and a branch point variant [34].

In the current study, we identified P/LP variants affecting noncoding regions, which would be missed with ES (Table 2). The interpretation of noncoding variants remains challenging, and in many cases, complementary methods based on enzyme activity determination, biomarker testing, and/or RNA analysis are necessary for variant classification and reporting, as demonstrated in our study. We recommend the use of this multi-omics approach to understand the functional consequences of noncoding and other genomic variants with unclear effect [25, 35], detected by GS serving as a diagnostic tool for precision medicine [31–34] .

A recent meta-analysis that included 154 studies performing genetic testing in patients with epilepsy detected that GS gave the highest diagnostic yield (48%), followed by ES (24%), gene panels (19%), and CMA (9%), supporting that GS should be prioritized as testing method for specific types of disorders [36–40].

Traditionally, the diagnostic odyssey is measured in years, which is then compared among patients and studies. However, this “crude” measure of the diagnostic odyssey in years might not accurately reflect the impact that the disease has had on patients or allow for comparisons between subjects of different ages [41].

In the current study, we used a “standardized” odyssey which allowed comparations between patients from different age groups. We found that patients with syndromic disorders have spent more than 75% of their life without a diagnosis, while for patients with neurologic and neuromuscular diseases the diagnostic odyssey was relatively short.

We found that pre-testing, specifically having karyotyping or CMA testing was associated with a longer time to reach a definitive diagnosis (*p* < 0.01). This is relevant since referring clinicians should be aware of the most efficient diagnostic tests for specific diseases. While in the past karyotyping and CMA testing were the recommended first tier testing methods, currents guidelines strongly recommend that ES/GS should be considered as a first- or second-tier test for patients with congenital anomalies, developmental delay, or intellectual disability [13].

The current limitations of the use of GS in the clinical practice include the difficulties in detecting SV (that do not lead a copy number change) but that can disrupt relevant sequences (especially relevant with short reads methodologies used for genome sequencing). Recent studies have shown that SVs can not only affect gene dosage but also modulate basic mechanisms of gene regulation. SVs are still mainly interpreted using the genetic dosage approach and general awareness of 3D position effects is low, leaving many patients undiagnosed [42]. We face difficulties in understanding the clinical relevance of noncoding variants. From the tecnical point of view, the detection of repeat expansions is still challenging. Finally, the lack of knowledge on the function of many genes is affecting the understanding of the relevance of the detected variants. Further analyses focusing in negative cases with similar clinical presentations can lead to the identification of new gene-disease relationships. As example, in three patients from this cohort, we identified a novel common variant in *SNUPN*. This helped to establish SNUPN deficiency as the genetic etiology of a previously unrecognized subtype of muscular dystrophy [43].

## Conclusion

This is the first LATAM study reporting the experience of the systematic use of diagnostic GS in patients with suspected RD. The diagnostic rate reached was close to 1 in 3.5 patients analyzed (28.3%), demonstrating the diagnostic utility of the test as reported in other populations. The diagnostic categories associated with metabolic, syndromic diseases, and neurocognitive disorders demonstrate the greatest technical robustness of GS in our cohort. The application of simultaneous multi-omics testing was valuable in assessing the clinical relevance of the detected variants. We anticipate that the current work will inspire the scientific and medical community to implement diagnostic GS as the preferred testing method in the diagnosis of RD in LATAM. Furthermore, we expect that governmental entities will recognize and accelerate the implementation of GS in the health care of patients with RD. Researchers and policymakers should continue to prioritize initiatives aimed at increasing diversity and representation in genomic databases and research studies to promote health equity and enhance our understanding of genetic contributions to disease.

## Supplementary information

Supplementary material

## Data Availability

Relevant information is listed in Supplementary Appendix 1–5. The datasets used and/or analyzed during the current study are available from the corresponding author on reasonable request.
