# Supplementary material for: Facing the challenges to shorten the diagnostic odyssey: first Whole Genome Sequencing experience of a Colombian cohort with suspected rare diseases
Source: Eur J Hum Genet. 2024 Jun 22;32(10):1327–37. doi: 10.1038/s41431-024-01609-8 (PMC11499989; doi:10.1038/s41431-024-01609-8)
Supplement: Supplementary file 1 — Supplementary material [file 41431_2024_1609_MOESM1_ESM.docx]

**SUPPLEMENTARY APPENDIX**

**Supplementary appendix 1.** The geographical distribution of the healthcare centers included in the Colombian rare genomes project.


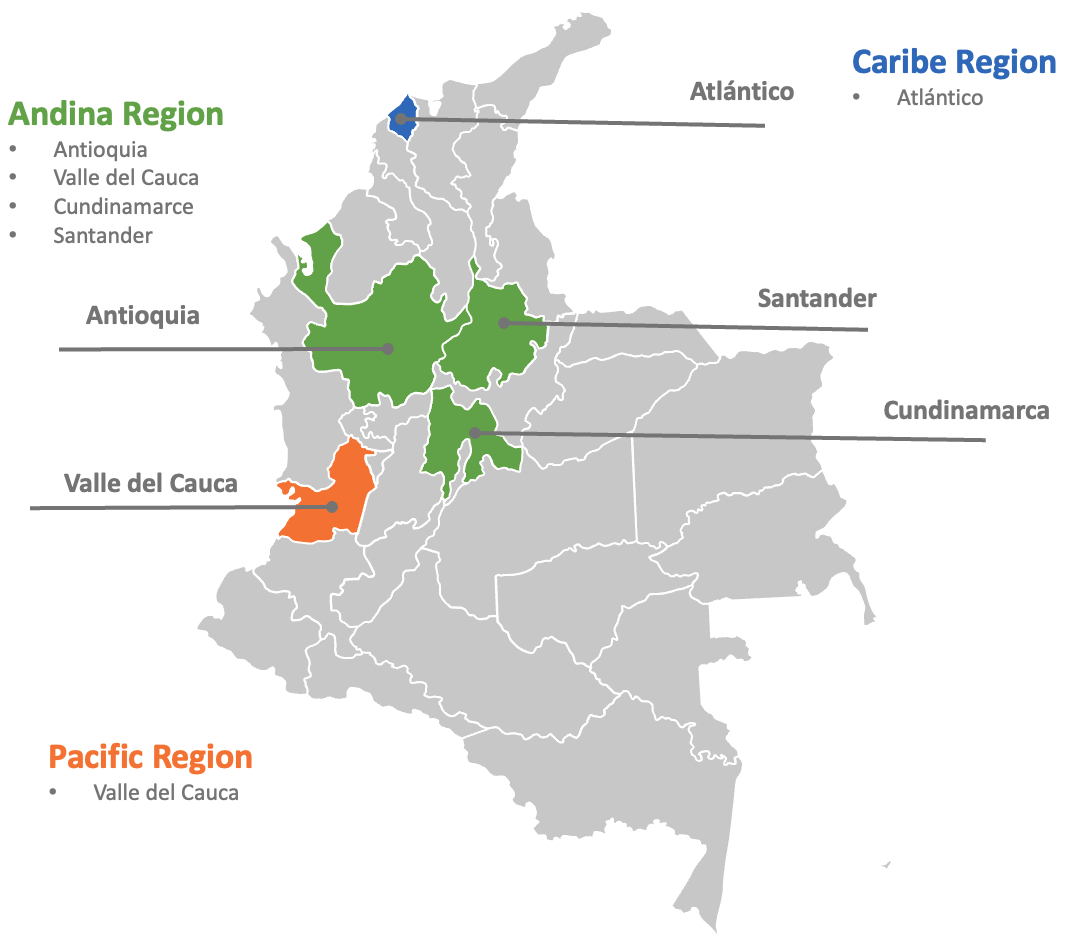


**Supplementary appendix 2:** Flowchart showing the inclusion of 501 patients in the Colombian genome sequencing project.

**
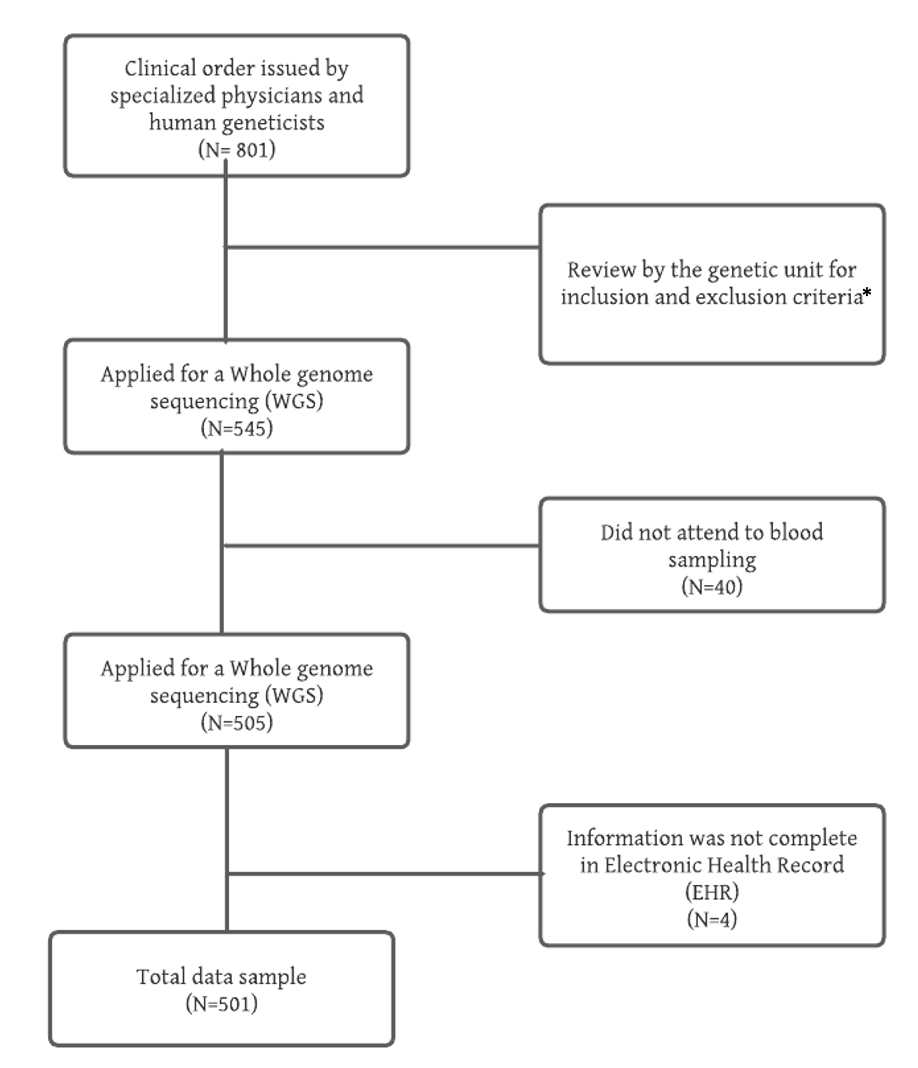
**

***Exclusion Criteria:** The following exclusion criteria was considered: 1) Having positive result with exome sequencing 2) Clinical presentation of isolated congenic anomalies (non-syndromic) as: mild cognitive disability, dyslipidemias, hyperelasticity syndromes, Marfan like syndromes, non-syndromic deafness, non-syndromic congenital heart disease, isolated major congenital anomalies, spina bifida, cleft lip and palate, clubfoot, hip dysplasia, isolated coloboma, limb reduction anomaly, ambiguous genitalia, hereditary cancer, single genetic coagulation disorders, recurrent miscarriage in the couple, deceased patients or any type of abortion or sample of ovular remains, or 3) Patients that did not attend the sampling appointment.

**Supplementary appendix 3A.** Genes with pathogenic/likely pathogenic variants (P/LP, left panel) and VUS (right panel) reported in at least two patients. Variants/genes reported as part of the carriership findings or ACMG secondary findings are not included in this graph.


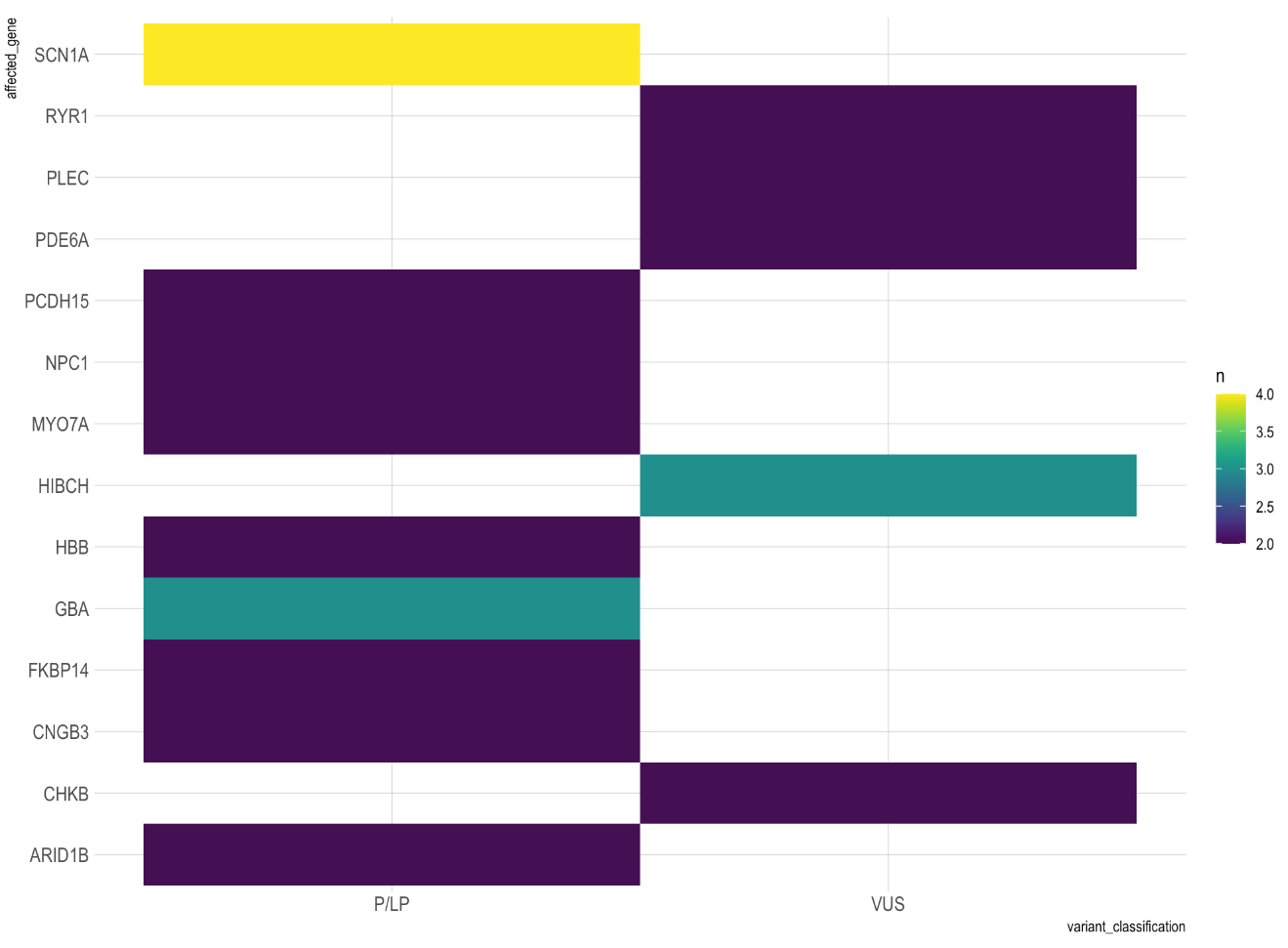


**Supplementary appendix 3B**. Genes from the carriership list and secondary findings list with pathogenic/likely pathogenic variants (P/LP, left panel) and VUS (right panel) reported in at least two patients.


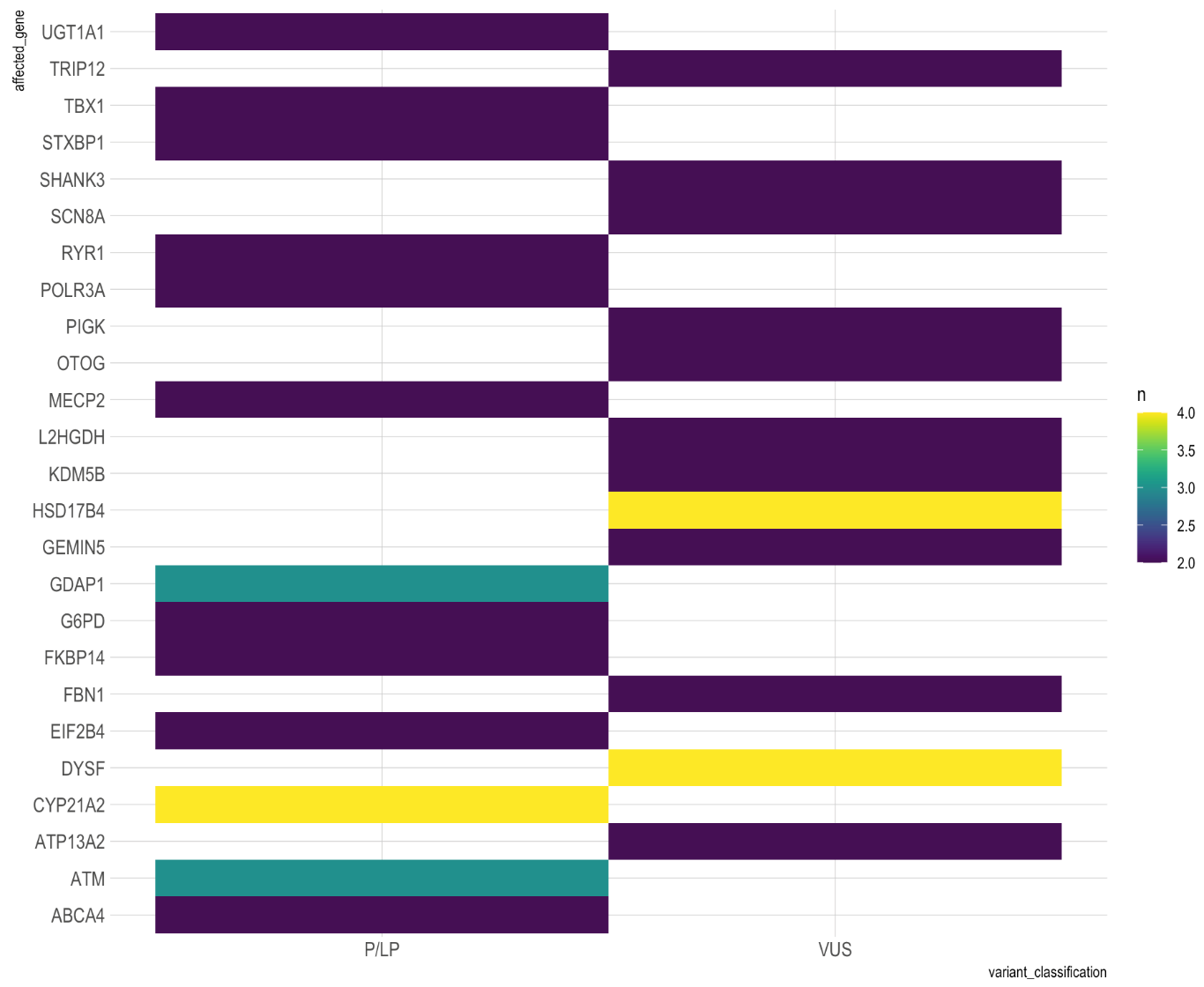


**Supplementary appendix 4**: Diagnostic odyssey in 142 patients that received a positive result, according to age. Significant differences were observed between younger and older patients, with the largest differences observed between the extreme age groups (0-5 years and 35-86 years, p-value < 0.001.


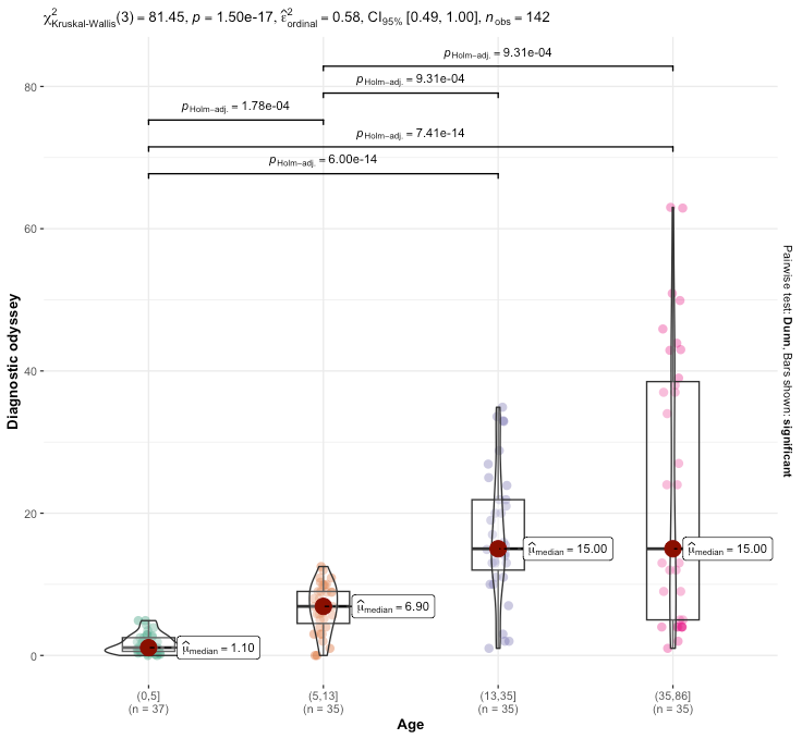


**Supplementary table 1.** Factors that could influence the test result. Only the total number of taxonomies showed borderline differences.

| **Characteristics** | **NEGATIVE**, N =263^1^ | **POSITIVE**, N = 144^1^ | **UNCLEAR** N = 180^1^ | **p-value**^2^ |
| --- | --- | --- | --- | --- |
| Age group |  |  |  | 0.15 |
| (0,6] | 79 (30%) | 38 (26,4%) | 51 (28,3%) |  |
| (6,12] | 55 (20,9%) | 29 (20,1%) | 49 (27,2%) |  |
| (12,19] | 51 (19,4%) | 18 (12,5%) | 22 (12,2%) |  |
| (19,45] | 47 (17,9%) | 31 (21,5%) | 32 (17,8%) |  |
| (45,86] | 31 (11,8%) | 28 (19,4%) | 26 (14,4%) |  |
| Parental consanguinity | 14 (6.1%) | 14 (11%) | 17 (11%) | 0.13 |
| Positive family history | 117 (45%) | 68 (48%) | 81 (46%) | 0.9 |
| Age | 12 (6, 24) | 14 (6, 38) | 11 (6, 30) | 0.5 |
| Age onset symptoms | 2 (0, 12) | 1 (0, 10) | 1 (0, 12) | 0.5 |
| Age at diagnosis | 12 (6, 24) | 14 (6, 38) | 11 (6, 30) | 0.5 |
| Number of HPO terms | 5 (3, 6) | 4 (3, 6) | 5 (3, 6) | 0.3 |
| Total number of taxonomies | 2.00 (2.00, 3.00) | 3.00 (2.00, 3.00) | 3.00 (2.00, 4.00) | **0.049** |

^1^ n (%); Median (Q1, Q3); n (%); ^2^ Fisher's exact test; Kruskal-Wallis rank sum test

**Supplementary appendix 5.** Non-standardized diagnostic odyssey, according to age ranges and groups of pathologies.

| **Disease category** | **(0,5] years old** | **(5,13] years old** | **(13,35] years old** | **(35,86] years old** |
| --- | --- | --- | --- | --- |
| Cognitive impairment | 0.4 (0.2,0.5) | 8.9 (7.8,9.4) | 14.7 (14.7,14.7) |  |
| Growth |  |  | 34.9 (34.9,34.9) | 44.5 (41.2,47.7) |
| Metabolic | 0.3 (0.2,0.4) | 8 (5.2,9.3) | 9.4 (2.5,17.9) | 24 (24,34) |
| Neurological | 1.5 (1.2,2.1) | 4 (3,5.8) | 11 (11,33) | 7 (4,12.8) |
| Neuromuscular | 0.6 (0.5,0.8) | 3 (3,3) | 15 (7,20) | 15 (9,37) |
| Syndromic | 1.9 (0.7,2.9) | 7.3 (5.7,9.9) | 15.5 (13,21.9) | 49.9 (45.9,62.9) |
| Visual |  | 6.9 (6.9,6.9) | 21 (21,21) | 12 (8,28) |
